# Takayasu arteritis: a cohort of Italian patients and recent pathogenetic and therapeutic advances

**DOI:** 10.1007/s10238-020-00668-7

**Published:** 2020-10-07

**Authors:** Franco Dammacco, Anna Cirulli, Annalisa Simeone, Patrizia Leone, Raffaele Pulli, Domenico Angiletta, Giuseppe Rubini, Alessandra Di Palo, Angelo Vacca, Rosanna Dammacco

**Affiliations:** 1 Department of Biomedical Sciences and Human Oncology, Medical School, Polyclinic, University of Bari Aldo Moro, Piazza Giulio Cesare, 11, 70124 Bari, Italy; 2 Radiology Department, Casa Sollievo della Sofferenza Hospital, San Giovanni Rotondo, Italy; 3 Department of Emergency and Organ Transplantation, Vascular and Endovascular Surgery Unit, Medical School, University of Bari Aldo Moro, Bari, Italy; 4 Department of Interdisciplinary Medicine, Nuclear Medicine Unit, Medical School, University of Bari Aldo Moro, Bari, Italy; 5 Department of Ophthalmology and Neuroscience, Medical School, University of Bari Aldo Moro, Bari, Italy

**Keywords:** Biologic agents, Imaging methods, Immunosuppressive therapy, Large-vessel vasculitis, Takayasu arteritis

## Abstract

Takayasu arteritis (TAK) is a rare granulomatous vasculitis of unknown etiology that mainly affects the aorta and its major branches. The aim is to describe the clinical features, diagnostic procedures, pathogenesis, and management of TAK in a longitudinal cohort of patients recruited within a single region of southern Italy. The cohort included 43 patients who were diagnosed with TAK and followed up according to a standard protocol, in a collaboration between four university tertiary referral centers and a regional hospital. Clinical and imaging classification criteria were those established by the American College of Rheumatology. Thirty-five patients (81.4%) were female, and the mean age at disease onset was 32.6 (range 16–54) years. Angiographic assessment of the vascular involvement allowed disease classification in five different types. Clinical features ranged from constitutional symptoms in the early inflammatory stage of the disease to cardiovascular ischemic symptoms in the late, chronic stage. Noninvasive imaging techniques were employed to assess the extent and severity of the arterial wall damage and to monitor the clinical course and response to therapy. Medical treatment, based on pathogenetic insights into the roles of humoral and cell-mediated immune mechanisms, included glucocorticoids mostly combined with steroid-sparing immunosuppressive agents and, in patients with relapsing/refractory disease, biologic drugs. Significant clinical and angiographic differences have been detected in TAK patients from different geographic areas. Patients with life-threatening cardiovascular and neurologic manifestations as well as sight-threatening ophthalmologic signs and symptoms should be promptly diagnosed, properly treated, and closely followed up to avoid potentially severe consequences.

## Introduction

Takayasu arteritis is a chronic, immune-mediated, large-vessel vasculitis that usually occurs in patients younger than 50 years of age and preferentially involves the aorta and its major branches. As a result, stenotic or occlusive lesions are a frequent feature of the disease, as are aneurysms and dissections [1–3]

A likely description of this arteritis dates back as far as 1830, when Rokushu Yamamoto described the case of a 45-year-old man with persistent high fever, who one year later developed impalpable upper limb and carotid pulses associated with remarkable weight loss and dyspnea, and died after 11 years of follow-up [4, 5]. But the first clear report of the disease was at the 1908 meeting of the Japanese Ophthalmological Society, when the ophthalmologist Mikito Takayasu described a 22-year-old female patient, observed 3 years earlier, who had a wreath-like anastomosis of retinal blood vessels around the optic disk [6]. Strikingly, at the same meeting another ophthalmologist, Yoshiakira Ohnishi, described “a case with similar changes in the fundus accompanying (an) impalpable pulse of the radial artery.” Consequently, this pathological condition was initially named “Takayasu-Ohnishi disease.”

However, such ophthalmologic findings are rarely encountered Based on the remarkable heterogeneity of its clinical and pathological features, several other names followed (“pulseless disease,” “aortic arch arteriostenosis syndrome,” “occlusive coagulant aortic syndrome,” “atypical coarctation of the aorta,” “aortic arch syndrome,” “obstructive productive arteritis”), but these are now considered reductive misnomers and the worldwide accepted name is Takayasu arteritis (TAK) [1, 7].

The overall annual incidence of TAK is 0.3–3.4 per million, and the prevalence is 0.9–40 per million. Most patients are women of child-bearing age living in Asian geographic areas; the male/female ratio ranges from 1:5 to 1:9 [3, 8, 9]. While TAK is a rare disease, over the last two decades it has been diagnosed with increasing frequency in patients of all ethnicities worldwide, in step with the improved awareness of this pathology and the extensive adoption of noninvasive imaging techniques [9–11].

The etiology of TAK is thus far undefined, but genetic, environmental, and autoimmune factors are likely to play an important role in its onset [12]. Although a link has been suggested with antigen-driven, immune-mediated processes including streptococcal infection-related disorders [13], syphilitic aortitis [14], and other pathogenic microbes or commensal microorganisms [15], an association between TAK and tuberculosis (TB) has been recognized on the basis of circumstantial evidence and isolated case reports [16, 17]. If unequivocally shown, this potential association would have important therapeutic implications, namely, the risk posed to patients by a worsening of active TB or the reactivation of latent TB following treatment with glucocorticoids and immunosuppressive drugs. In particular, the administration of tumor necrosis factor-*α* (TNF-*α*) inhibitors, often chosen to treat patients with recurrent or refractory TAK (discussed below), would be expected to increase the risk of TB development by up to 25-fold [18]. However, systematic and careful reviews of the literature indicate that the risk is not higher in TAK patients than in patients with other rheumatic diseases treated with TNF-α inhibitors [19, 20]. These observations are considered proof of concept that the relationship between TAK and TB is epiphenomenal rather than causal, although the clinical picture of TB arteritis should be considered in the differential diagnosis of TAK.

The aim of the present study was to evaluate the clinical, diagnostic, and therapeutic features of TAK in a longitudinal cohort of patients recruited within a single region of southern Italy. Recent insights into the pathogenesis of TAK and the potential treatment implications are discussed.

## Patients and methods

This retrospective, observational study was based on a cohort of 43 patients diagnosed with TAK from 2006 to 2018 and followed up in a collaboration between four tertiary referral centers of the University of Bari Medical School and a general hospital of the Apulia region.

Each patient underwent complete clinical and laboratory assessments, performed according to a standard protocol. Medical records were carefully reviewed to ensure that the patients met at least three of the following classification (five clinical and one imaging) criteria established by the American College of Rheumatology (ACR) [7]: (1) development of symptoms or findings related to TAK at age ≤ 40 years; (2) claudication of the extremities, prevalently the upper limbs, defined as the development or worsening of fatigue and discomfort in the muscles of one or more extremity while in use; (3) decreased brachial artery pulse, with reduced pulsation of one or both brachial arteries; (4) systolic blood pressure difference between arms of > 10 mmHg; (5) bruit audible on auscultation over one or both subclavian arteries or the abdominal aorta; (6) arteriographic narrowing or occlusion of the entire aorta, its primary branches, or of the large arteries in the proximal upper or lower extremities, not due to arteriosclerosis, fibromuscular dysplasia or similar causes, and with changes usually focal or segmental. The reported sensitivity and specificity of these criteria are 90.5% and 97.8%, respectively [7].

Based on the angiographic assessment of vascular involvement, five types of TAK have been defined [21, 22]. In patients with type I, the aortic arch and its major branches are primarily involved. Type IIa affects the ascending aorta, aortic arch, and its branches. Type IIb involves the ascending aorta, aortic arch and its branches, and the thoracic descending aorta. In type III, both the thoracic descending aorta and the abdominal aorta, with or without the renal arteries, are involved. Type IV involves the abdominal aorta and/or the renal arteries, and in type V the inflammatory process extends to the entire aorta and its branches. The additional designation of C( +) or P( +) identifies involvement of the coronary or pulmonary arteries, respectively.

Disease severity was established according to the criteria of the Japanese Research Committee for Intractable Vasculitis [3], which include the following: (a) grade I, medically treatable without the use of glucocorticoids; (b) grade II, medically treatable with the use of glucocorticoids (GC); (c) grade III, uncontrollable disease activity despite glucocorticoids or invasive treatment (endovascular or open surgery); (d) grade IV, surgical or intensive medical treatment to address a life-threatening complication (aortic regurgitation, aneurysm, renal artery stenosis, ischemic heart disease, pulmonary infarction); (e) grade V, severe organ failure (congestive heart failure, myocardial infarction, respiratory failure, cerebrovascular accident, cataract, renal failure, mental disorder).

A Mantoux tuberculin skin test was performed in 27 out of 43 patients (62.8%) and found to be weakly positive in 2 of them (7.4%), whereas an interferon-Ɣ release assay (Quantiferon TB Gold) tested negative in all of them and a chest X-ray revealed no abnormalities compatible with latent tuberculosis. In addition, disease activity was defined based on the occurrence of two or more of the following items: (a) systemic signs or symptoms not ascribable to other conditions; (b) erythrocyte sedimentation rate (ESR) > 30 mm/h and/or a C-reactive protein (CRP) level > 8 mg/L after the exclusion of infections or malignancy as well as other vasculitides and autoimmune diseases; (c) signs or symptoms of vascular insufficiency; d) extension of existing lesions or the appearance of new lesions, detected by imaging studies. Patients in whom the clinical and laboratory features of active disease resolved in the absence of new vascular lesions detected on sequential imaging studies were considered in remission. A remission lasting for at least 6 months in patients receiving a daily regimen of < 10 mg prednisone was defined as sustained. Finally, patients with disease relapse were those experiencing a flare of disease activity following a remission phase [23].

Statistical analysis was carried out by calculation of the mean, standard deviation, and median for continuous variables, and the number/percent of patients for categorical variables. Differences in the proportion between groups were assessed by the Mantel–Haenszel Chi-squared test or Fisher’s exact test.

All procedures conformed with the tenets of the 1964 Helsinki Declaration and its later amendments. The University of Bari Ethics Committee approved the study and, based on its retrospective case record review design, waived the need for the written informed consent of the patients to study enrollment.

## Results

### Study cohort

Our study cohort of 43 patients comprised 35 females (81.4%) and 8 males (18.6%), with a F/M ratio of 4.4:1. The mean age at onset was 32.6 (range, 16–54) years [32.2 (range, 16–49) years in females and 34.2 (19–54) years in males]. In the 13 patients (27.9%; 11 females and 2 males) > 40 years of age at the time of diagnosis, all of the other diagnostic criteria of TAK were met but not the ACR criteria for giant cell arteritis (GCA) [24]. Overall, the median diagnostic delay was 23 (range, 9–33) months. The mean length of follow-up for the whole cohort was 51 (9–84) months.

### Clinical findings

The clinical features of the patients at diagnosis are described in Table 1. In 5 patients (4 females and 1 male, 11.6%) who were initially asymptomatic, TAK was diagnosed while searching for the cause of an accidentally discovered arterial hypertension (3 patients), in the course of a health examination before starting a new job and in association with obtaining a life insurance policy (1 patient each). In the remaining 38 patients, variable combinations of the signs and symptoms listed in Table 1 were observed. The early inflammatory phase of the disease was typically characterized by non-specific constitutional symptoms, usually with a relapsing/remitting clinical course, whereas in the late, chronic, “pulseless” stage, the clinical picture was dominated by cardiovascular ischemic symptoms reflecting the narrowing, occlusion or dilation of the aorta and/or its main branches [9, 25].

**Table 1:** Chief complaints at diagnosis in a cohort of patients with Takayasu arteritis

Signs and Symptoms		Total (43 pts) No (%)	Female (35 pts) No (%)	Male (8 pts) No (%)	*p*-value
Asymptomatic	Casually discovered arterial hypertension	5 (11.6)	4 (11.4)	1 (12.5)	1.000
Constitutional	Persistent low-grade fever, fatigue, general ill feeling, anorexia, weight loss, night sweats, nausea and vomiting	38 (88.4)	32 (91.4)	6 (75)	0.2276
Musculoskeletal	Arthralgia/arthritis, myalgias, joint stiffness	9 (20.9)	8 (22.8)	1 (12.5)	1.000
Cardiovascular	Tachycardia and palpitation, precordial pain, new-onset hypertension, brachial pulse deficit with blood pressure discrepancy > 10 mm Hg, aortic valve insufficiency, upper or lower limb claudication, pain and tenderness on palpation over the carotid bifurcation, vascular bruits	36 (83.7)	30 (85.7)	6 (75)	0.5970
Pulmonary	Paroxysmal or exertional dyspnea, hemoptysis, cyanosis, pulmonary hypertension	5 (11.6)	4 (11.4)	1 (12.5)	1.000
Ophthalmologic	Amaurosis fugax, monocular or binocular blurred vision, reduction of visual acuity, ocular pain, metamorphopsia	16 (37.2)	11 (31.4)	5 (62.5)	0.125
Neurologic	Headache, numbness, dizziness, syncope, transient ischemic attacks	11 (25.6)	8 (22.8)	3 (37.5)	0.401
Renal	Renovascular hypertension, proteinuria, hematuria	9 (20.9)	7 (20)	2 (25)	1.000
Dermatologic	Erythema nodosum, livedo reticularis, toe ulcers	4 (9.3)	3 (8.6)	1 (12.5)	1.000
Gastrointestinal	Abdominal pain, nausea, vomiting, diarrhea	3 (6.9)	3 (8.6)	0	1.000

**Fig. 1:**
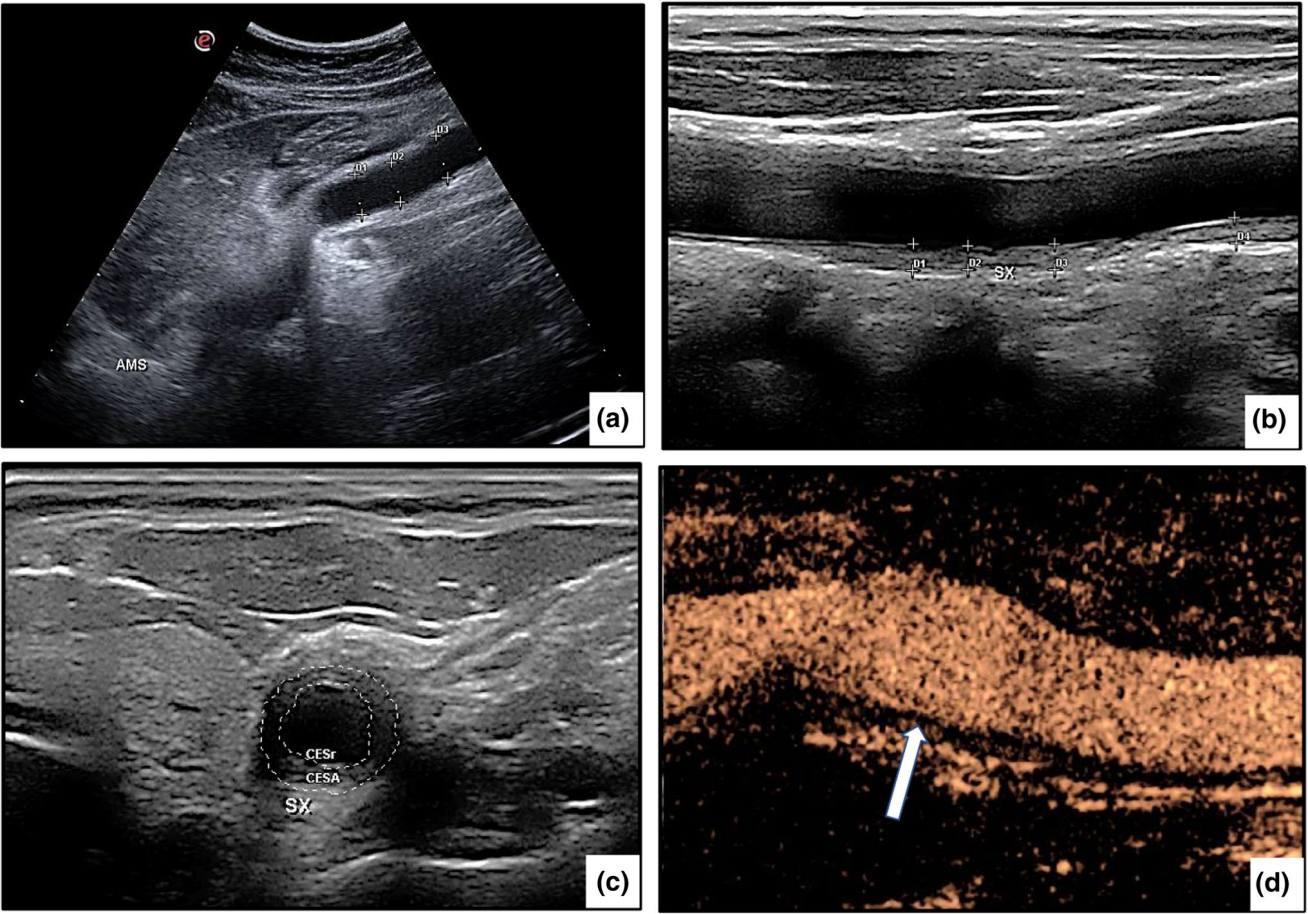
**a** A 33-year-old woman with type III Takayasu arteritis complained of abdominal pain, nausea, vomiting, and diarrhea. A B-mode ultrasound showed the rare finding of superior mesenteric artery aneurysm. The diagnosis of “superior mesenteric artery syndrome” was considered. **b** B-mode ultrasound image of the right common carotid artery in a 27-year-old man diagnosed with type IIa Takayasu arteritis. Longitudinal B-mode ultrasound shows mid-echoic, homogeneous circumferential wall thickening (“macaroni sign”). The thickness of the intima–media is between 1.3 and 2.0 mm (normal value ≤ 0.9 mm). **c** A transverse scan of the same vessel shows marked, concentric, hypoechoic wall thickening that reduces the lumen by > 50%. **d** Contrast-enhanced ultrasound of the carotid artery allows to detect a high uptake of microbubbles, indicating active disease

Ocular manifestations were detected in 16 of our patients (37.2%), usually late in the course of the disease, and were frequently responsible for severe visual deterioration. These patients are described in detail in a separate report (Dammacco R et al., submitted for publication). Musculoskeletal and neurological features were less frequent, but not rarely were the presenting symptoms of the disease. Renovascular hypertension, often associated with proteinuria and microhematuria, was a prominent manifestation, detected in 7 of the 9 patients with type V TAK and in 2 patients with type IV TAK. Dermatologic findings, mostly erythema nodosum, and abdominal symptoms indicative of gastrointestinal involvement (Fig. 1A) were detected in a minority of patients. A comparative analysis of the clinical findings according to sex showed that constitutional and cardiovascular features were more frequent in female patients, whereas ocular manifestations were diagnosed in a larger number of male patients. The differences, however, were not statistically significant (Table 1).

### Laboratory findings

The ESR and the CRP level were increased in 38 (88.4%) and 34 (79.1%) patients, respectively. Hypochromic normocytic anemia (15/43: 34.9%), leukocytosis (19/43: 44.2%), and thrombocytosis (27/43: 62.8%) were detected in patients with disease in the active phase at the time of diagnosis. An additional laboratory finding was polyclonal hypergammaglobulinemia, seen in 53.5% of the patients. None of the 43 patients had abnormalities in renal function. HLA genetic studies were available in 17 of the 43 patients, comprising the subset enrolled from 2009 onward. An association with the HLA-B*52 allele was clearly established in 5 of these patients (29.4%) but in only 4 of 50 (8%) healthy, unrelated Caucasian volunteers from the same geographic area who served as controls (*p* < 0.02). However, given the low number of typed patients, a relationship between HLA-B*52 and the severity of TAK or the presence of complications could not be firmly established.

### Imaging studies

The extensive application of noninvasive imaging techniques is essential to detect arterial wall damage, including arterial stenosis or dilation, to assess the extent and severity of vascular tree involvement, and to monitor the clinical course of the disease and the response to treatment [11, 26, 27]. Our patients were observed over a period of about 13 years, during which time they underwent several noninvasive imaging studies, including at diagnosis and at periodic follow-up examinations.

The most widely used of these techniques was high-resolution color Doppler ultrasonography (CDUS), performed in all patients. Among the findings were a homogeneous, hypo-echogenic, circumferential thickening of the arterial wall affecting to a variable extent and in variable combinations the carotid, subclavian, axillary, and vertebral arteries, either mono- or bilaterally, as well as the abdominal aorta. In several instances, ultrasonography showed a characteristic circumferential thickening of the intima–media complex, a pattern referred to as the “macaroni sign” [12, 28] (Fig. 1B, C) and considered pathognomonic for TAK [29]. Other advantages of CDUS, in addition to its ability to assess intima–media thickness, are the absence of radiation exposure and the easy repetition of the imaging study [30]. However, the technique is operator dependent and unable to accurately image the aortic arch and descending aorta. A more reliable assessment is provided by contrast-enhanced ultrasonography (CEUS), which is more sensitive than serum measurements of acute-phase reactants, such as ESR and CRP, in the monitoring of disease activity and the response to therapy (Fig. 1D) [31, 32].

**Fig. 2:**
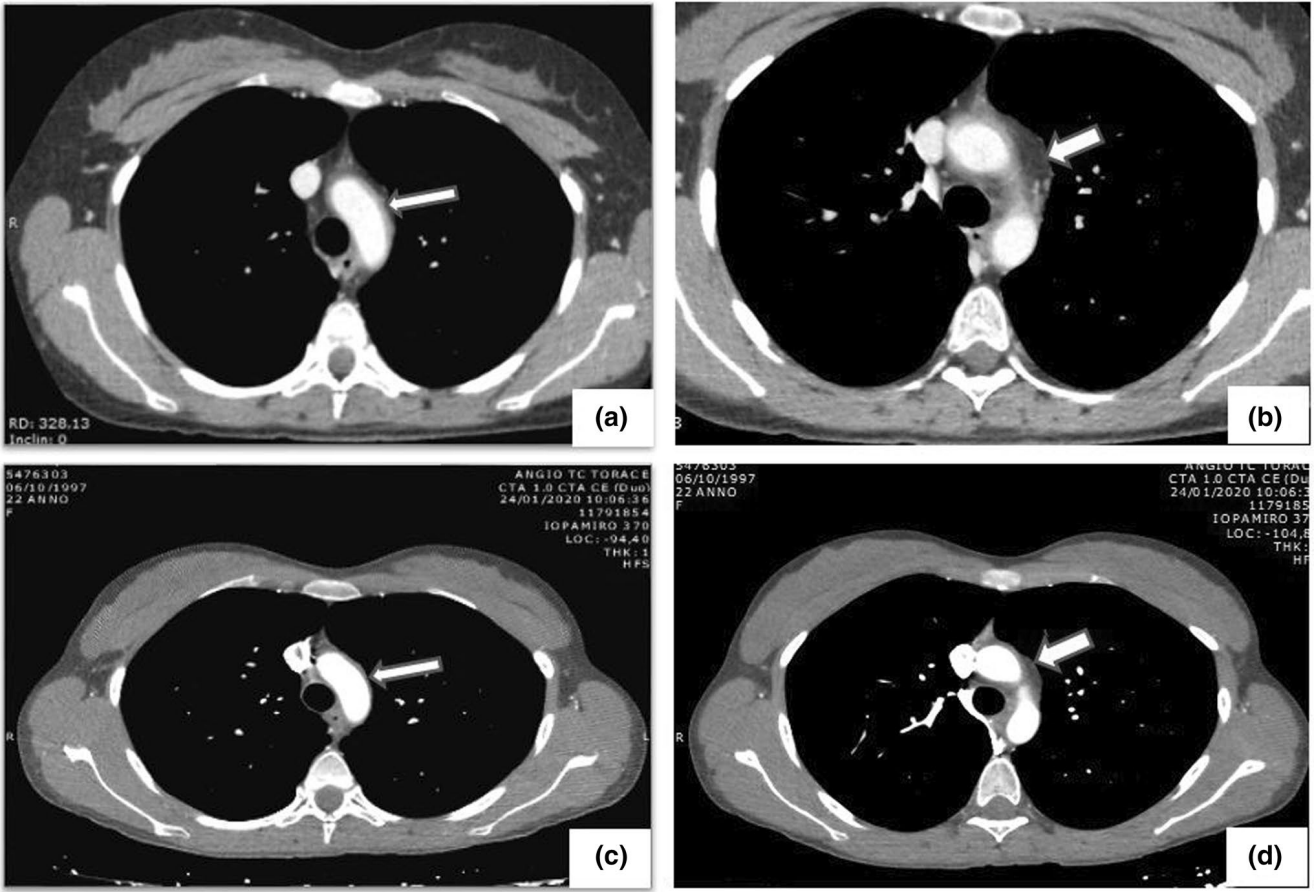
**a**, **b** Angio-CT with intravenous iodinated contrast medium: note the circumferential thickening and enhancement of the aortic arch wall (arrows). **c**, **d** Following medical treatment and using the same sequence scans, both wall thickening and enhancement are clearly reduced

**Fig. 3:**
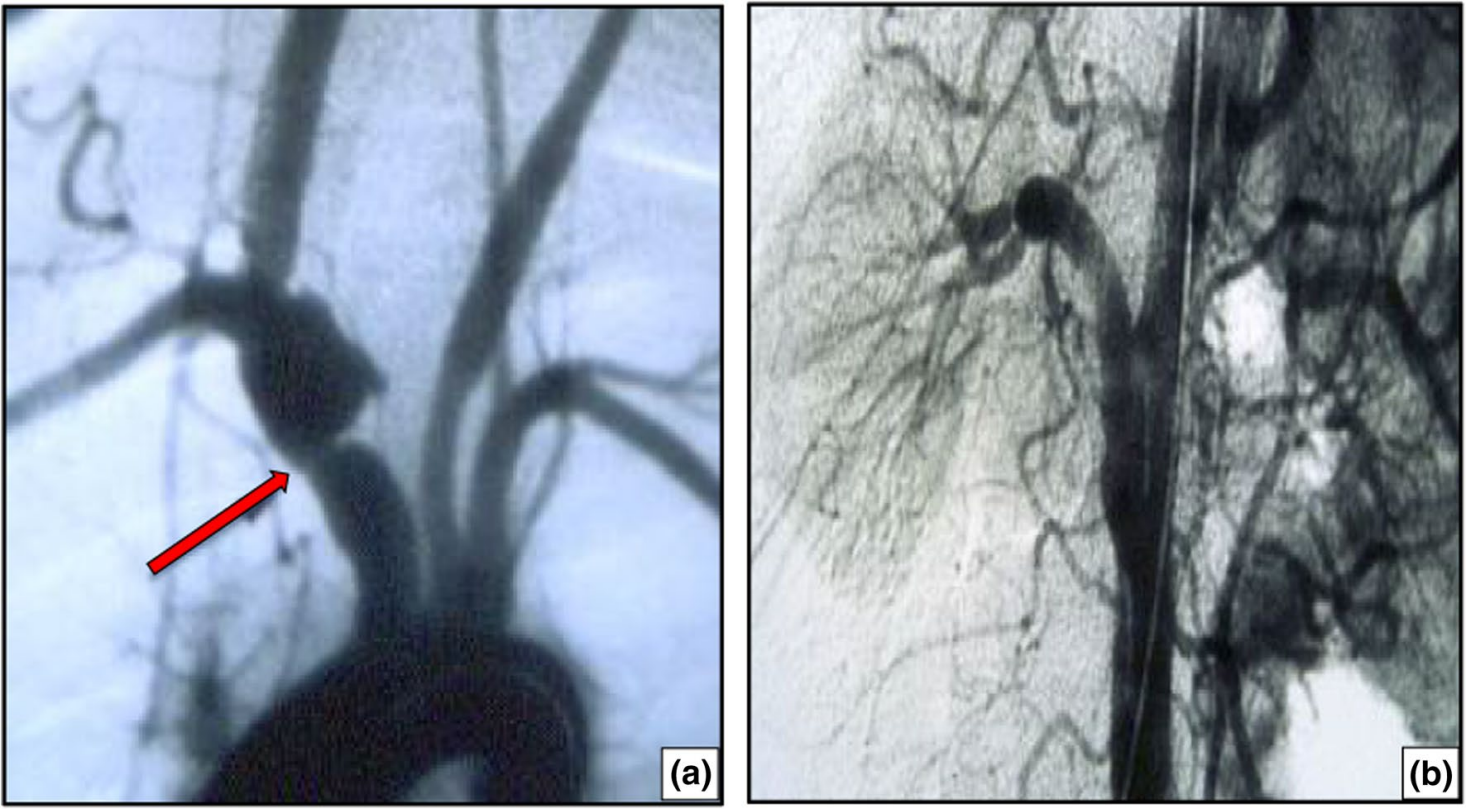
**a** Stenosis of the anonymous trunk with post-stenotic dilation in a 21-year-old young lady with type I Takayasu arteritis. **b** A 34-year-old male patient was diagnosed with type V Takayasu arteritis, sub-occlusive stenosis of the right renal artery, and arterial hypertension. Following aorto-renal bypass surgery, blood pressure was normalized and anti-hypertensive medication was no longer required

**Fig. 4:**
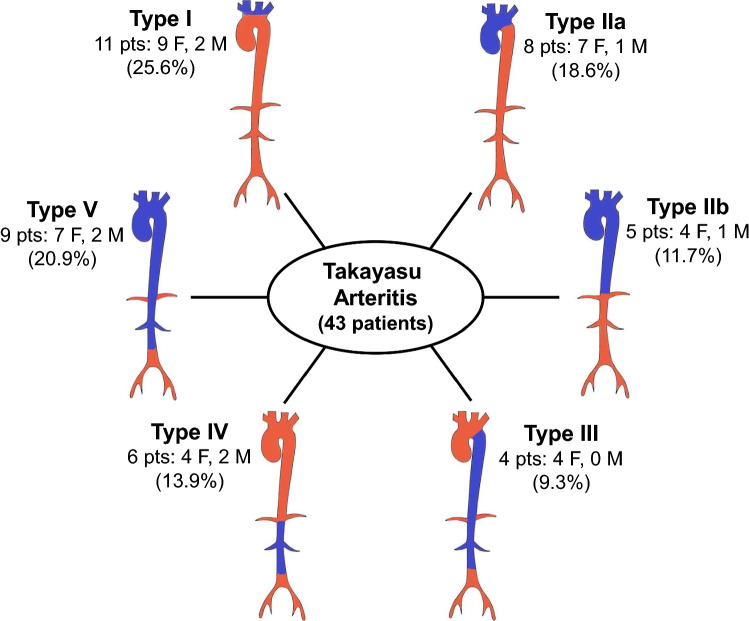
Angiographic classification of Takayasu arteritis according to the involved arteries in our cohort of 43 patients [15]

Computed tomography angiography (CTA) was performed in all patients and provided a clear image of the vessels involved as well as the lumen abnormalities, including stenotic and aneurysmal lesions (Figs. 2 and 3). Based on the angiographic patterns, the disease in each patient was classified according to the five above-described types (Fig. 4). Types I and V were the most common, diagnosed in 25.6% and 20.9% of the patients, respectively, followed by type IIa (18.6%), type IIb (13.9%), type IV (11.6%), and type III (9.3%). The limitations of CTA are the use of iodinated contrast agent, administered intravenously, and the exposure of patients to a high level of radiation.

**Fig. 5:**
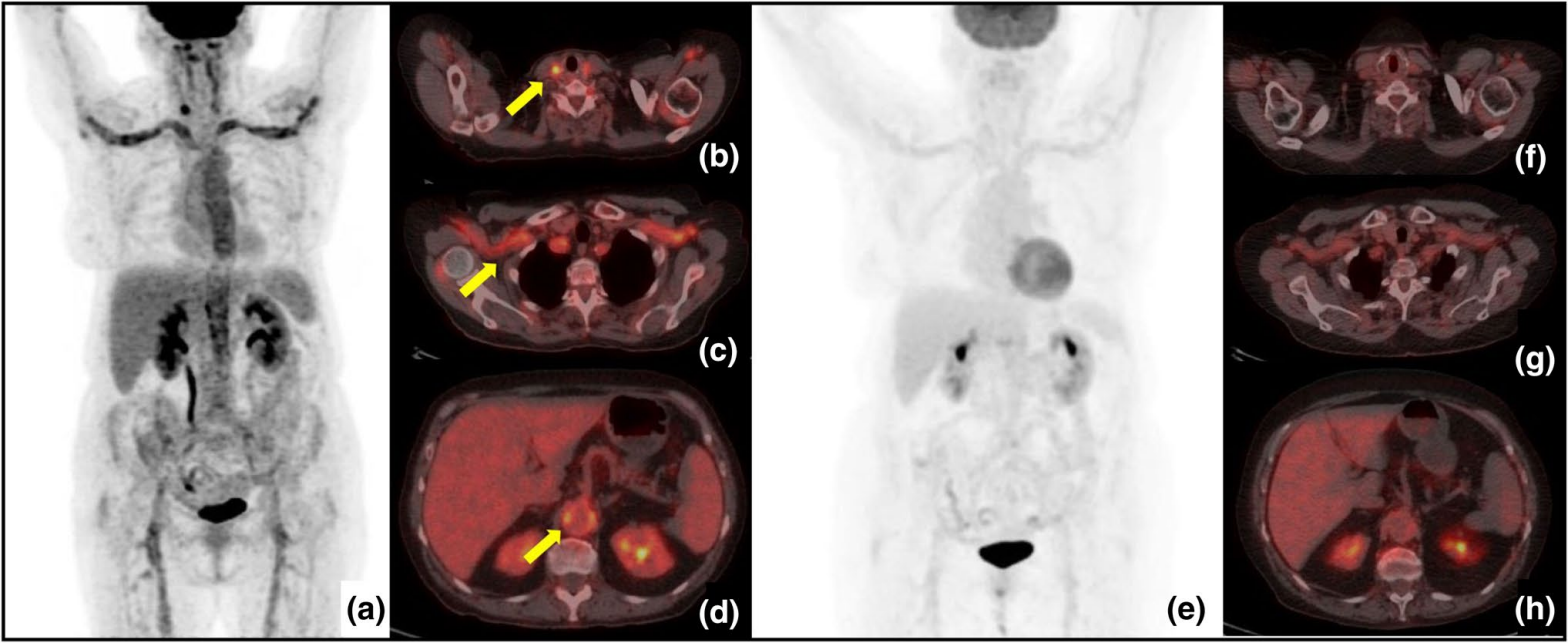
^18^F-FDG PET/CT in a 42-year-old female patient with type V Takayasu arteritis who complained of fatigue, weight loss, fever, and severe headache. Maximum intensity projection (MIP) (**a**) and axial fused images showed increased ^18^F-FDG uptake (yellow arrows) in the aorta and its main branches, particularly in carotid (**b**) and subclavian (**c**) arteries and in the distal abdominal aortic wall (**d**). After 6 months of therapy, follow-up PET/CT images show clearly decreased uptake in the large arteries (E–H)

^18^F-Fluorodeoxyglucose positron emission tomography/computed tomography (FDG-PET/CT) is being used with increasing frequency due to its high sensitivity and the ability to detect the sites, extent, and severity of vessel inflammation. Within our cohort, this angiographic tool was employed in 12 patients (27.9%) at diagnosis and provided clear images of high sensitivity (Fig. 5). However, the limitations of FDG-PET/CT include a rapid decrease in sensitivity in patients treated with GC for 1–2 weeks prior to the examination, high-level radiation exposure, and the possible misinterpretation of the lesions as atherosclerosis [33, 34].

**Fig. 6:**
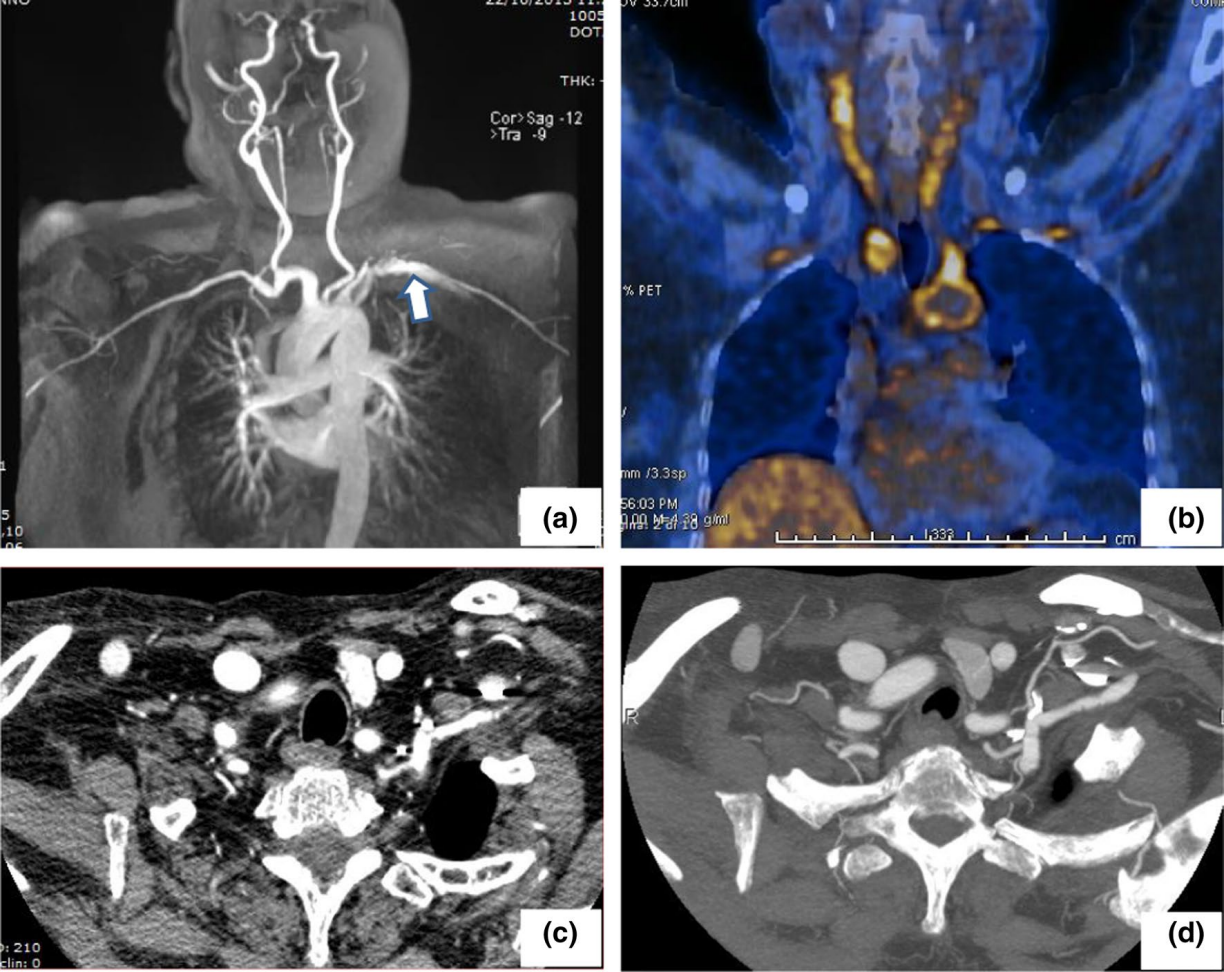
**a** Angio-MR with intravenous paramagnetic contrast medium shows a moderate ectasia and an irregular contour of the proximal tract of left subclavian artery (arrow). **b** By ^18^FDG-PET/CT an increased uptake of the radiopharmaceutical can be seen at the level of the neck vessels and thoracic aorta. **c** Following therapy, CT with intravenous iodinated contrast medium reveals a reduced irregularity of the left subclavian wall and a more homogeneous gauge. **d** The same CT with maximum intensity projection (MIP) algorithm

Magnetic resonance angiography (MRA), performed in 9 of our patients (20.9%), provided detailed images of the vascular tree (Fig. 6). The further advantages of MRA include reliable information on luminal and mural changes, the examination of vessels in any suitable plane, the option to obtain serial assessments over time given the lack of exposure to ionizing radiation (a particularly valuable feature in young patients), and the short time required for image generation. The disadvantages include difficulty in visualizing small branch vessels and vessel wall calcifications, and the high cost of the technique [35, 36].

Clinical and imaging-based evaluations often show a variable correlation with the disease course [37]. In the assessment of TAK, MRA and FDG-PET/CT often provide unique and complementary information. Although MRA is more suitable to establish the extent of the disease, FDG-PET/CT is preferred if the goal is to assess vascular activity.

### Medical treatment

**Fig. 7:**
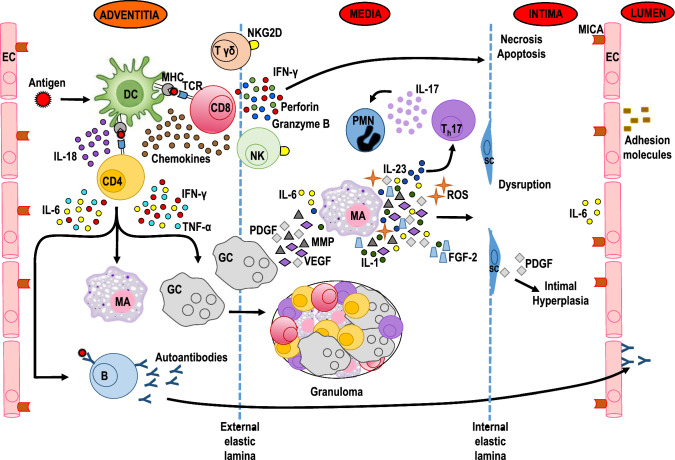
Hypothetical immunopathogenesis of Takayasu arteritis. A so-far-unknown antigen is presented to CD4 and CD8 T cells recruited from the vasa vasorum by major histocompatibility complex (MHC) molecules expressed on dendritic cells (DC) resident in the adventitia. DC release interleukin (IL)-18 and chemokines, which attract and retain additional T cells. Activated CD4 T cells trigger B cell activation, which results in autoantibody production and the apoptosis of vascular endothelial cells (EC). They also produce interferon (IFN)-γ, IL-6, and tumor necrosis factor (TNF)-α. These cytokines induce the activation and differentiation of macrophages and giant cells which, along with lymphocytes, form granulomas. Activated macrophages and giant cells release IL-23, IL-6, IL-1, reactive oxygen species (ROS), matrix metalloproteinases (MMP), fibroblast growth factor (FGF)-2, platelet-derived growth factor (PDGF), and vascular endothelial growth factor (VEGF), which together cause oxidative injury, disruption of the elastic laminae, intimal hyperplasia and neoangiogenesis. The differentiation of IL-17-producing T cells (Th17 cells) by IL-23 results in the attraction and activation of polymorphonuclear neutrophils (PMN) in the vessel wall, thus contributing to inflammation and vascular damage. Activated CD8 and γδ T cells as well as natural killer (NK) cells release IFN-γ, perforin, and granzyme-B, which promote the apoptosis and necrosis of smooth muscle cells, with consequent injury to the intimal layer. NK cells and γδ T cells are also able to trigger a strong cytotoxic response against vascular EC mediated by natural killer group 2 member D/major histocompatibility class I-related chain A (NKG2D-MICA) interactions [19]

To understand the rationale of the medical therapies commonly employed in TAK, it is necessary to consider the immunopathogenesis underlying disease onset. Both humoral and cell-mediated immune mechanisms are involved in the pathogenesis of TAK [25, 38]. The persistent activation of immune cells and continued release of proinflammatory cytokines in the aortic adventitia and media result in granuloma formation, fibrosis, scarring and vascularization of the media, disruption of the internal elastic lamina, intimal hyperplasia, and occlusion, with or without thrombosis [39–41]. Autoantibodies targeting human heat-shock protein, endothelial cells, and annexin V (a protein that induces apoptosis in vascular endothelial cells) have been detected in TAK patients [42–44], but whether they have a pathogenetic role or are an epiphenomenon is unclear. The complex pathogenetic interactions that characterize TAK are depicted schematically in Fig. 7.

Given the inflammatory nature of TAK, and as recommended by the European League Against Rheumatism (EULAR) [45], GC are considered the mainstay of treatment. Their early, usually high-dose administration is essential to control vascular inflammation, achieve clinical remission, and prevent or limit end-organ damage due to ischemia. As summarized in Table 2, all our patients received GC, with an initial daily dose of prednisone ranging from 0.5 to 1 mg/kg that after three weeks was gradually tapered to a maintenance daily dose of 5–10 mg, in step with the attenuation or regression of signs and symptoms as well as the normalization of the ESR and CRP level.

**Table 2:** Medical treatment in our cohort of 43 patients with Takayasu arteritis

Drugs and dosages	Patients (*n*)	%
At diagnosis and throughout the active phase: 43 patients*		
Glucocorticoids (GC): 1 mg/kg/day per os	2	4.6
GC: 1 mg/kg/day per os plus Cyclophosphamide: 1.5 mg/kg per os	2	4.6
GC: 0.5–1 mg/kg/day per os plus Azathioprine	13s	30.2
GC: intravenous pulses of 1 g daily for 3 days followed by GC: 1 mg/kg/day per os plus Methotrexate: 15 mg weekly subcutaneously	1	2.3
GC: 0.5–1 mg/kg/day per os plus Methotrexate: 15 mg weekly subcutaneously	25	58.1
With relapsing/refractory disease: 12 patients*		
GC: 0.5–1 mg/kg/day per os plus Tocilizumab: 4 mg/kg intravenously, every 2 weeks for 3 months and every 4 weeks for another 3 months	7	58.3
GC: 1 mg/kg/day per os plus Methotrexate: 15 mg weekly subcutaneously plus Adalimumab: 40 mg subcutaneously, every 2 weeks for 3 months and every 4 weeks for another 3 months	2	16.6
GC: 1 mg/kg/day per os plus Methotrexate: 15 mg weekly subcutaneously Plus Rituximab: 375 mg/m^2^ once a week for 4 weeks followed by two 5-monthly infusions	3	25.0

^*^In all patients, the glucocorticoid dosage was maintained for 3 to 5 weeks and then gradually tapered

Since our patients were recruited in a time frame of about 13 years, their treatment was inevitably heterogeneous. In the first two patients, with type I and IIa disease, respectively, the clinical course was mild and treatment consisted of GC only. However, in the remaining 41 patients an immunosuppressive agent was regularly added to achieve a GC-sparing effect and to reduce the risk of relapse. Within this group, GC were combined with cyclophosphamide in 2 patients and with azathioprine in 13 patients. GC in combination with methotrexate (MTX) were prescribed in 60% of the patients, including 1 patient with type IIb TAK in whom the oral administration of GC was preceded by three intravenous pulses of methylprednisolone because of the occurrence of persistently high fever and a particularly severe clinical picture (Table 2).

Bone protection with bisphosphonates was prescribed to all but 6 patients (in whom these drugs were contraindicated) plus calcium and vitamin D, as appropriate. All patients receiving MTX were also prescribed a folate supplement to reduce the mucosal, gastrointestinal, hepatic, and hematologic side effects. Anti-platelet therapy with aspirin was given to 12 patients, 9 of whom were > 40 years of age at diagnosis. The remaining 13 patients had a moderate lymphopenia and subnormal levels of circulating IgG following long-term GC therapy and were thus treated with three doses of trimethoprim/ /sulfamethoxazole per week to prevent *Pneumocystis jirovecii* pneumonia.

Remission was achieved in 38 patients (88.4%) in a median of 4.9 (range, 3.3–8.6) months. In 25 of them (58.1%) the remission was sustained, whereas 5 patients (11.6%) had a partial response. One or more relapses were recorded in 12 patients (27.9%), occurring in the first 6 months from the time of the first remission in 9 of them. Of the 7 patients with relapse who had been treated with GC plus tocilizumab, 4 experienced a sustained remission and 3 a partial response. The two patients treated with GC plus MTX plus adalimumab achieved a transient remission, with relapses occurring 3 and 5 months after the discontinuation of adalimumab but still during tapered doses of GC and 10 mg MTX/week. Combination therapy consisting of GC in combination with MTX and the anti-CD19 monoclonal antibody rituximab (RTX) was employed in 3 patients with refractory TAK: two of them were considered to be in sustained remission at the end of treatment and one was lost to follow-up 6 months after the first administration of RTX.

The most common complications of TAK were arterial hypertension (Fig. 3B), with or without renal arterial stenosis (13 patients: 30.2%), and aortic valve regurgitation (7 patients: 16.3%). Visual symptoms and impaired visual acuity caused by steroid-induced cataracts or consequent to restenosis after a patency procedure developed in 4 patients (9.3%) and 2 patients (4.6), respectively; they are described in detail elsewhere (Dammacco R et al. submitted). Three patients died 35, 41, and 57 months after their diagnosis, as a consequence of cerebral hemorrhage, aortic dissection, and myocardial infarction, respectively.

## Discussion

An early diagnosis of TAK remains challenging in the initial “pre-pulseless” phase of active inflammation, due to the predominance of vague, insidious, constitutional symptoms (Table 1) and a lack of specific clinical and/or laboratory features that raise suspicion. Vascular signs such as arterial hypertension, pulse and systolic blood pressure inequalities between arms, and upper-limb claudication are early features in at least one-third of patients [3, 25, 46], but may be initially unrecognized. When the diagnosis of vasculitis is suspected, it is therefore suggested that family doctors measure blood pressure in both arms and legs. Although an increased level of tissue inhibitor of metalloproteinases (TIMP-1) has been proposed as a laboratory biomarker in the diagnosis of TAK, it is poorly specific, often occurring also in psoriasis, liver fibrosis, and cancer [47].

The difficulty in the early diagnosis of TAK, especially in patients with remitting/relapsing clinical symptoms, explains why diagnostic delays ranging from a few months to approximately 4 years have been reported in major retrospective cohort studies [23, 48, 49]. Thus, the diagnostic delay of 9–33 months in our cohort was not unusual. However, a late diagnosis and the consequent late establishment of a suitable treatment inevitably result in more severe vascular involvement. This is especially the case in children, who at the time of diagnosis have significantly more aortic and renal artery involvement, and a higher frequency of arterial hypertension than is true for adults [50].

Nonetheless, a similar initial diagnostic difficulty can occur in adults. The first of the six criteria developed by the ACR for the classification of TAK was onset at age ≤ 40 years [7]. Consequently, an indefinite number of older patients complaining of persistent low-grade fever, night sweats, and arthralgia have mistakenly been diagnosed with GCA rather than late-onset TAK. In our cohort, 28% of the patients were older than 40 years at diagnosis, and similar demographics have been reported in other studies [3, 46, 51]. The criterion of age ≤ 40 years was eventually withdrawn [52], replaced by the definition developed by the Revised International Chapel Hill Consensus Conference, which recognized TAK as a large-vessel vasculitis with an onset that usually occurs before the age of 50 years, a major distinction from GCA, whose onset usually occurs after age 50 [2].

The age extension for the diagnosis of TAK inevitably raises the question whether TAK and GCA should be considered skewed phenotypes within the spectrum of the same disease [53, 54], but the answer is as yet unknown. Strong similarities between TAK and GCA include several shared clinical features, laboratory evidence of systemic inflammation, the frequent imaging finding of involvement of the aorta and its branches, the indistinguishable granulomatous nature of the lesions on biopsy, the female predominance, and the pathogenetic role of Th‐1 and Th-17 cells. Nonetheless, the two diseases differ in their genetic structure, geographic prevalence, and epidemiological characteristics. In addition, 40–60% of GCA patients also have polymyalgia rheumatica [55], which is consistently absent in TAK patients, and while the extra-cranial branches of the external carotid arteries are involved in GCA, the primary branches of the aorta are typically affected in TAK. Overall, until the etiologies of the two diseases are definitively determined, or their shared etiology is confirmed, it seems reasonable to believe that, in spite of the mentioned similarities, TAK and GCA should be considered as distinct conditions [53, 54].

The angiographic classification of TAK differs significantly in patients from different geographic areas worldwide. In general, type I seems to be the most common type in European patients and is seen in adults more often than in children [56], whereas types V and IV prevail in children and non-Europeans [8, 57, 58]. In terms of a sex-based prevalence, type I has been reported more frequently in females and type V in males, at least in Japan [3, 51]. Obviously, the variable angiographic features are not simply ethnically or genetically related nor sex- or age-related, but inevitably result in different clinical manifestations depending on the vessels that are prevalently or exclusively involved by the granulomatous process [12, 59]. Among our patients, 11 (25.6%) had type I and 9 (20.9%) type V disease, roughly consistent with the findings of an Italian poly-center study of 104 patients with TAK [48], although the latter authors did not adopt the angiographic classification established by the 1994 International TAK Conference [21, 22]. In the last decade, the increasing use of noninvasive imaging techniques has remarkably contributed to a better definition of the site and extension of the vascular damage and has considerably shortened the delay between symptom onset and diagnosis.

TAK is usually progressive in its course: the early active “pre-pulseless” phase is gradually replaced by a late occlusive phase, characterized by the protean clinical features summarized in Table 1. Among them, both the cardiovascular and neurologic manifestations and the vision-impairing ophthalmologic signs and symptoms may be life-threatening and must be promptly diagnosed and treated and then closely followed up to avoid dreadful consequences for the patients. In particular, systemic hypertension frequently associated with renal artery stenosis can result in death due to hypertensive cardiovascular and cerebrovascular accidents [23, 48, 60]. Similar complications occurred in 3 of our patients and led to their death, as a consequence of cerebral hemorrhage, aortic dissection, and myocardial infarction, respectively. Ophthalmologic manifestations, on the other hand, were relatively frequent in our cohort (16 patients: 37.2%) and resulted in impairment of the best corrected visual acuity, which was severely reduced in 20 eyes: reduced to counting fingers and hand motion in 2 and 2 eyes, respectively; and to no light perception in 1 eye (Dammacco R et al., submitted). The risk of irreversible impairment of visual acuity due to a late diagnosis highlights the importance of a careful ophthalmological assessment in all patients with TAK, both at diagnosis and during follow-up.

Several studies have addressed the immunopathogenetic mechanisms underlying the onset of TAK [9, 15, 25, 61]. A summary of the current understanding of the roles likely played by cellular and humoral immunity in TAK is provided in Fig. 7. A deeper insight into the pathogenesis of this disease is crucial to establishing a rational therapeutic approach. Although the initial eliciting agent remains unidentified, it is apparently able to trigger an immune reaction that targets the walls of large vessels and eventually results in severe vascular damage and ultimately stenosis or aneurysm.

The primary role played by inflammation and autoimmunity in the pathogenesis of TAK is supported by the positive clinical response induced in the majority of patients by GC and immunosuppressive drugs. However, in a minority of patients with relapsing/refractory TAK, pro-inflammatory cytokines such as TNF-*α*, IL-6, IL-17, and IFN-γ likely exert a pathogenic role and should therefore be therapeutically addressed. This is the goal of TNF-*α* inhibitors, such as etanercept, infliximab, adalimumab, and certolizumab [8, 62–64], as well as anti-interleukin (IL)-6 receptor monoclonal antibodies such as tocilizumab [65–68], which are increasingly being administered. However, controlled clinical studies in a suitable number of patients are required to allow definitive conclusions about the efficacy of these agents.

Encouraging results in terms of a higher remission rate and rapid control of refractory disease have been reported in 40–60% of TAK patients [8, 67]. Nonetheless, the benefit among those treated with GC was not sustained when the dose was ≤ 30 mg/day, even in patients with low disease activity [69], and vascular progression developed in at least one-third of the patients treated with biological drugs, either during treatment or after drug withdrawal [61, 63, 70]. In our own, thus far limited experience, 2 patients treated with GC plus MTX plus adalimumab achieved a transient clinical response, but their disease relapsed a few months after the discontinuation of adalimumab, despite continued treatment with a low dose of GC and a maintenance dose of MTX of 10 mg/week. Of the 7 patients with disease relapse that was treated with GC plus tocilizumab, 4 had a sustained remission and 3 a partial response, including a retardation of the angiographic progression.

The pathogenetic involvement of B cells in active TAK is supported by several observations. First, numerous B cells have been detected immunohistochemically in the inflamed arterial adventitia of aortic wall samples from TAK patients [71]. Second, the frequency of B cell subsets, including newly generated plasmablasts and memory B cells, is higher in the peripheral blood of TAK patients than in that of healthy donors [72]. Third, as already mentioned, autoantibodies targeting human heat-shock protein, endothelial cells, and annexin V have been detected in TAK patients, although their pathogenic role remains undefined [42–44]. Overall, these findings suggest a role for B cell-depleting therapy in TAK patients. Indeed, the results so far obtained in small numbers of patients, including 3 of our own patients, support the use of RTX as a potentially effective third-line therapeutic option in patients with relapsing TAK resistant to combinations of GC plus immunosuppressive factors and biologic drugs [73, 74].

## Conclusions

Prospective, controlled, clinical trials with long-term follow-up will be required to assess the efficacy, safety, and the exact therapeutic role of the various combinations of immunosuppressive and biologic agents in TAK patients, the optimal duration of treatment, effective drug-tapering strategies and the identification of predictors of a favorable response. Given the rarity of TAK, the establishment of an International Registry of Takayasu Arteritis is strongly encouraged to facilitate the collection of suitable cohorts of patients.

Admittedly, our study had the following limitations: (1) its retrospective nature, resulting in a lack of uniformity in the diagnostic and therapeutic procedures; (2) the relatively small cohort of 43 patients, compared to the much larger numbers of patients recruited in poly-center or nationwide epidemiological cohort studies [3, 17, 39, 42]; (3) the revascularization of the involved arteries through reconstructive surgery or less invasive endovascular interventions, including angioplasty balloon or graft replacement stents, carried out by different vascular surgeons in our own university hospital or in extra-regional expert centers.

However, the strengths of this study include: (1) the detailed collection of data, made possible through a carefully implemented collaboration among four specialized units of the same university and one hospital unit of the same geographic region; (2) clinical assessments made by the same clinicians throughout patient recruitment and follow-up, which reduced the risk of unwanted variability; (3) the lengthy follow-up, which ranged from 9 to 84 months and in 25 of the 43 study patients (58.1%) exceeded 5 years.
